# A Theoretical and Simulation Analysis of the Sensitivity of SiNWs-FET Sensors

**DOI:** 10.3390/bios11040121

**Published:** 2021-04-15

**Authors:** Yi Yang, Zicheng Lu, Duo Liu, Yuelin Wang, Shixing Chen, Tie Li

**Affiliations:** 1 Science and Technology on Microsystem Laboratory, Shanghai Institute of Microsystem and Information Technology, Chinese Academy of Sciences, Shanghai 200050, China; hanshu@mail.sim.ac.cn (Y.Y.); snowlzc@mail.sim.ac.cn (Z.L.); ylwang@mail.sim.ac.cn (Y.W.); 2 University of Chinese Academy of Sciences (UCAS), Beijing 100190, China; 3 State Key Laboratory of Crystal Materials, Institute of Novel Semiconductors, 27 South Shanda Road, Jinan 250100, China; liuduo@sdu.edu.cn

**Keywords:** silicon nanowires (SiNWs), field effect transistor (FET), sensor, sensitivity, cross-section

## Abstract

Theoretical study and software simulation on the sensitivity of silicon nanowires (SiNWs) field effect transistor (FET) sensors in terms of surface-to-volume ratio, depletion ratio, surface state and lattice quality are carried out. Generally, SiNWs-FET sensors with triangular cross-sections are more sensitive than sensors with circular or square cross-sections. Two main reasons are discussed in this article. Firstly, SiNWs-FET sensors with triangular cross-sections have the largest surface-to-volume ratio and depletion ratio which significantly enhance the sensors’ sensitivity. Secondly, the manufacturing processes of the electron beam lithography (EBL) and chemical vapor deposition (CVD) methods seriously affect the surface state and lattice quality, which eventually influence SiNWs-FET sensors’ sensitivity. In contrast, wet etching and thermal oxidation (WETO) create fewer surface defects and higher quality lattices. Furthermore, the software simulation confirms that SiNWs-FET sensors with triangular cross-sections have better sensitivity than the other two types of SiNWs-FET sensors under the same conditions, consistent with the theoretical analysis. The article fully proved that SiNWs-FET sensors fabricated by the WETO method produced the best sensitivity and it will be widely used in the future.

## 1. Introduction

The increasing demand for the detection of biological and chemical molecules, such as tumor markers [1,2,3] and volatile organic compounds (VOCs) [4], has become imperative in the past several years. Ultrahigh sensitivity is essential for the detection of these targets. Currently, thin film sensors and nanosensors are widely adopted for this mission because of their advantages in sensitivity [5,6,7]. Among these sensors, SiNWs-FET sensors have received increasing attention as they have ultrahigh sensitivity, which is necessary for the detection of these samples [8,9,10,11]. There are many factors, such as debye length [12], surface binding sites [13] and molecular affinities [14], that affect the sensitivity of SiNWs-FET sensors. Larger debye length, more surface binding sites and stronger molecular affinities lead to better sensitivity.

**Figure 1:**
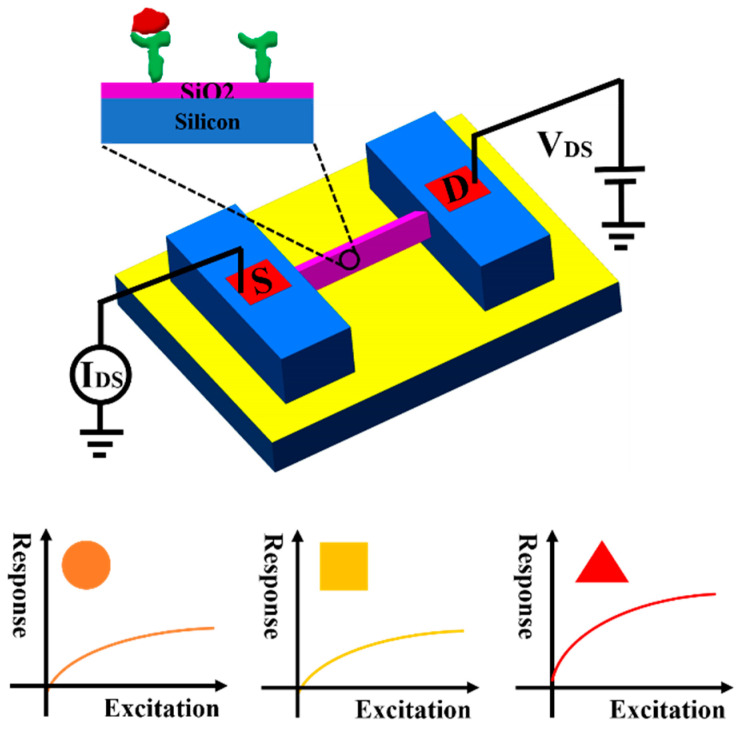
The sensitivity of three types of SiNWs-FET sensors with circular, square and triangular cross-sections.

In recent years, a succession of methods have been developed to fabricate various SiNWs. Generally, the fabrication methods of SiNWs can be divided into three types: CVD (based on the vapor-liquid-solid (VLS) mechanism) [15,16], EBL (following dry etching) [17], and wet etching followed by thermal oxidation (WETO) [18,19,20]. In contrast to the VLS and EBL methods, SiNWs-FET sensors fabricated by WETO method have remarkable advantages such as the controllability of the whole process and the capability of wholesale manufacture [21]. Additionally, the cross-sections of the SiNWs fabricated by these three methods are circular, square and triangular, respectively, which seriously affects the performance of devices [22,23,24,25,26,27]. Here, we compared the sensitivity of the SiNWs-FET sensor fabricated by our WETO method with the others by theoretical analysis and software simulations. Typically, the sensitivity of sensors is defined as: *sen =* ∆*I/I*_0_, and we compared these three types of SiNWs-FET sensors by analyzing the factors that affect ∆*I* or *I*_0_. Notably, the surface-to-volume ratio, depletion ratio, surface defects and the quality of the lattice are selected in this article to discuss the sensitivity of SiNWs-FET sensors. We found the triangular cross-sectioned SiNWs-FET sensors fabricated by WETO method are more sensitive than SiNWs-FET sensors fabricated by the other two methods when they have the same feature size, as shown in Figure 1.

## 2. Theoretical Analysis

We analyzed the sensitivity of SiNWs-FET sensors with three different cross-sectional shapes (circle, square and triangle), along the same feature size *W*. In order to accurately express the sensitivity of the SiNWs-FET sensors, the derivation process started from the definition of the current and the expression of sensitivity is described as follows [10]:

(1) $$sen=\frac{∆I}{I_{0}}=\frac{∆n}{n_{0}+n_{1}+n_{2}}+\frac{∆S_{sec}}{S_{sec}}+\frac{∆S_{sec}\cdot ∆n}{S_{sec}∆\left(n_{0}+n_{1}+n_{2}\right)}$$

where *q* is the elementary charge, ∆*I* is the variation of current, *I*_0_ is the reference current, ∆*S_sec_* is depleted area in a cross-section of silicon nanowire, *S_sec_* is the cross-sectional area of silicon nanowire, ∆*n* is the variation of carrier concentration, *n*_0_ is the initial carrier concentration, *n_1_* is the carrier concentration brought about by crystal defects and *n_2_* is the carrier concentration brought about by surface defects, respectively.

**Figure 2:**
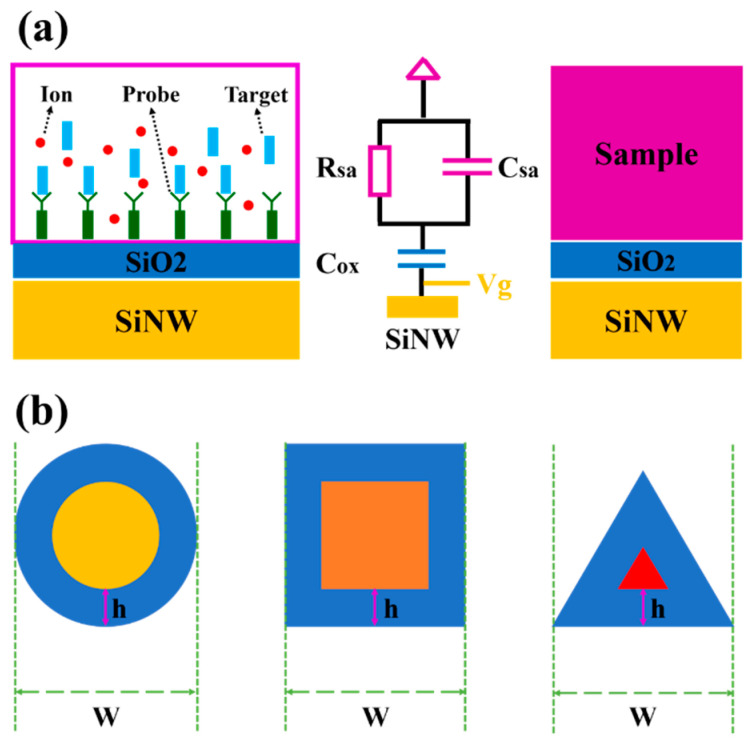
(**a**) The schematic of the sensitive mechanism during the process of target detection and the equivalent circuit model for SiNWs-FET sensors. (**b**) Cross-section view of the depleted area of the three types of silicon nanowire with the same depletion depth *h*.

The sensing mechanism of SiNWs-FET sensors during target detection, the equivalent circuit model for SiNWs-FET sensors and its diagram are depicted in Figure 2a. The variation of carrier concentration in silicon nanowire is given by [28]

(2) $$∆n=\frac{Q_{S}\cdot S_{sur}}{q\cdot V}$$

where *Q_s_* is the density of charge carried by the sensing target on the silicon nanowire surface, *S_sur_* is the surface area exposed to visual field of silicon nanowire, *q* is the elementary charge and *V* is the volume of the silicon nanowire. Therefore, we can obtain the relationship between the sensitivity and the surface-to-volume ratio, so the sensitivity can be described as

(3) $$sen\propto Q_{S}\cdot \frac{S_{sur}}{V}$$

In one case, when the target concentration is extremely low, we can assume that the magnitude of charge carried by target is far from saturation, namely *Q_R_*, and the sensitivity of SiNWs-FET sensors is given by

(4) $$sen\propto \frac{Q_{R}}{S_{sec}L}$$

where *L* is the length of silicon nanowire. When these three types of silicon nanowires have same length *L*, the cross-sectional area of the silicon nanowire is a critical factor. With the same feature size *W*, the cross-sectional areas are listed in Table 1.

As described in the Equation (4), if $S_{sec}^{tri}<S_{sec}^{cir}<S_{sec}^{squ}$, the relationship of these three SiNWs-FET sensors’ sensitivity is $sen_{tri}>sen_{cir}>sen_{squ}$. As a result, the SiNWs-FET sensor with the triangular cross-section has the best sensitivity under this condition.

In the other case, when the target concentration is high, the density of the surface charge is saturated. We supposed that these three types of SiNWs-FET sensors had the same density of surface charge $Q_{s}$. Under the same length and feature size of silicon nanowire, we calculated the surface-to-volume ratio of these three types of silicon nanowires, and the results are listed in Table 2.

As described in the Equation (3), because ${\left(\frac{S_{sur}}{V}\right)}_{squ}\leq {\left(\frac{S_{sur}}{V}\right)}_{cir}<{\left(\frac{S_{sur}}{V}\right)}_{tri}$, $sen_{tri}>sen_{cir}\geq sen_{squ}$ and we obtained the same conclusion as before.

As described in Equation (1), the sensitivity of SiNWs-FET sensors is directly affected by the depletion ratio and the expression can be simplified as follows:

(5) $$sen\propto \frac{∆S_{sec}}{S_{sec}}$$

As shown in Figure 2b, we assume that the depletion depth is identical among these three kinds of SiNWs-FET sensors and denoted as *h*. The depletion ratio can be analyzed and the results are listed in Table 3.

Owing to the fact that the depletion ratio of SiNWs-FET sensors with triangular cross-section is larger than the others, it has the best sensitivity among these three types SiNWs-FET sensors.

Different from the surface-to-volume ratio and depletion ratio, which affects SiNWs-FET sensors’ sensitivity through impact on *∆I*, more surface defects and a poor quality lattice lead to higher background noise, which also have a great influence on the SiNWs-FET sensors’ sensitivity [29].

**Figure 3:**
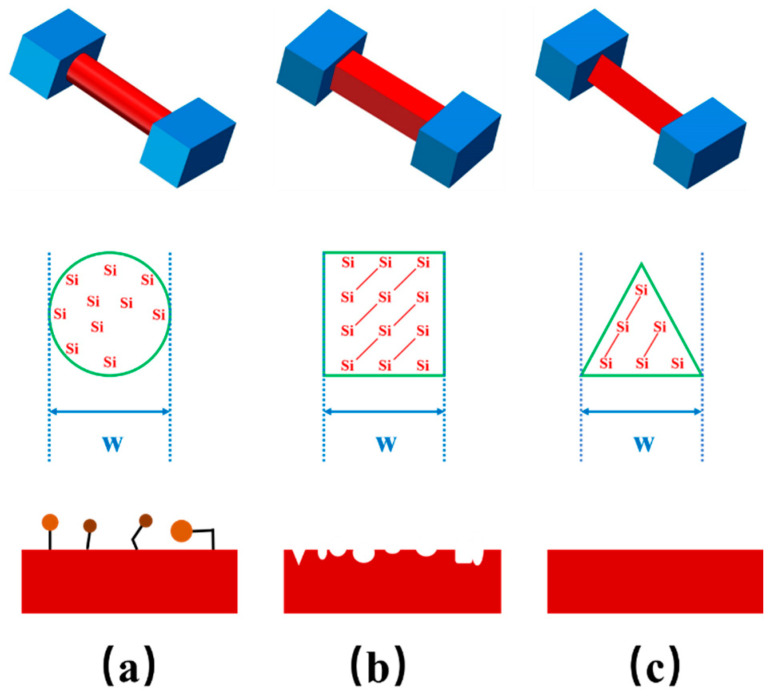
Three types of SiNWs-FET sensors and diagrammatic sketch of surface states and lattice quality with their cross-sections being (**a**) circle, (**b**) square, (**c**) triangle, respectively under the same feature size W.

Silicon nanowires grown by CVD based on VLS mechanism are usually amorphous in nature, which means that there exist numerous dangling bonds on the surfaces [30]. The dangling bonds will generate extra carrier concentration *n_1_* (as shown in Equation (1)) and therefore lead to extra current *I*_1_ *= n*_1_*qsv*, which makes the dark current higher than the others. As shown in Figure 3a, there are many chemical groups suspended on the surface of the silicon nanowire grown by CVD based on VLS mechanism. Next, due to the characteristics of EBL, SiNWs-FET sensors fabricated by EBL followed by dry etching, which is shown in Figure 3b, are also less sensitive. This is because the dry etching method could partially damage the surface of the silicon nanowire [31], which also increases the dark current by *I*_2_ *= n*_2_*qsv*. Therefore, due to the larger density of the surface defects, SiNWs-FET sensors fabricated by these two methods have higher background signals than SiNWs-FET sensors fabricated by the WETO method. The probability of electron occupied surface defects is given by [32,33,34,35,36].

(6) $$f_{SD}\left(E_{SD}\right)=\frac{1}{1+\frac{1}{g}\cdot \exp\left(\frac{E_{SD}-E_{F}}{k_{0}T}\right)}$$

where *E_SD_* is the surface state energy, *g* is the degeneracy of ground state, *E_F_* is Fermi level, *k_0_* is the Boltzmann’s constant, *T* is the thermodynamic temperature, respectively. Therefore, the per unit area number of the surface defects occupied by electrons is given by

(7) $$n_{2}=\int_{E_{SD}}^{E_{SD}^{\prime }}\frac{N_{SS}\left(E\right)dE}{1+\frac{1}{g}\cdot \exp\left(\frac{E-E_{F}}{k_{0}T}\right)}$$

where *N_SS_* is the per unit area number of surface states in unit-energy interval for the energy level *E*, *E_SD_* and $E_{SD}^{\prime }$ are the upper and lower limits of surface states energy level in band gap, respectively.

The density of the surface states of the silicon nanowire fabricated by CVD based on the VLS mechanism or EBL and dry etching is larger than the density of the silicon nanowire fabricated by WETO [37], thus we can obtain the relationship between the density of the surface states of the three types of silicon nanowires as follows: *N_SS_*(triangle) < *N_SS_*(square) and *N_SS_*(triangle) < *N_SS_*(circle). Therefore, SiNWs-FET sensors with triangular cross-section have fewer surface states and lower dark current.

In contrast, EBL and dry etching give rise to partly damaged surfaces of silicon nanowire, thus the SiNWs-FET sensors have poor sensitivity. Additionally, due to bad lattice quality, silicon nanowires grown by the CVD method also have worse sensitivity, which is affected by the chemical groups on the sensor surface. In summary, SiNWs-FET sensors with triangular cross-section fabricated by WETO brings fewer surface defects and thus exhibits better sensitivity.

## 3. Simulation Verification

**Figure 4:**
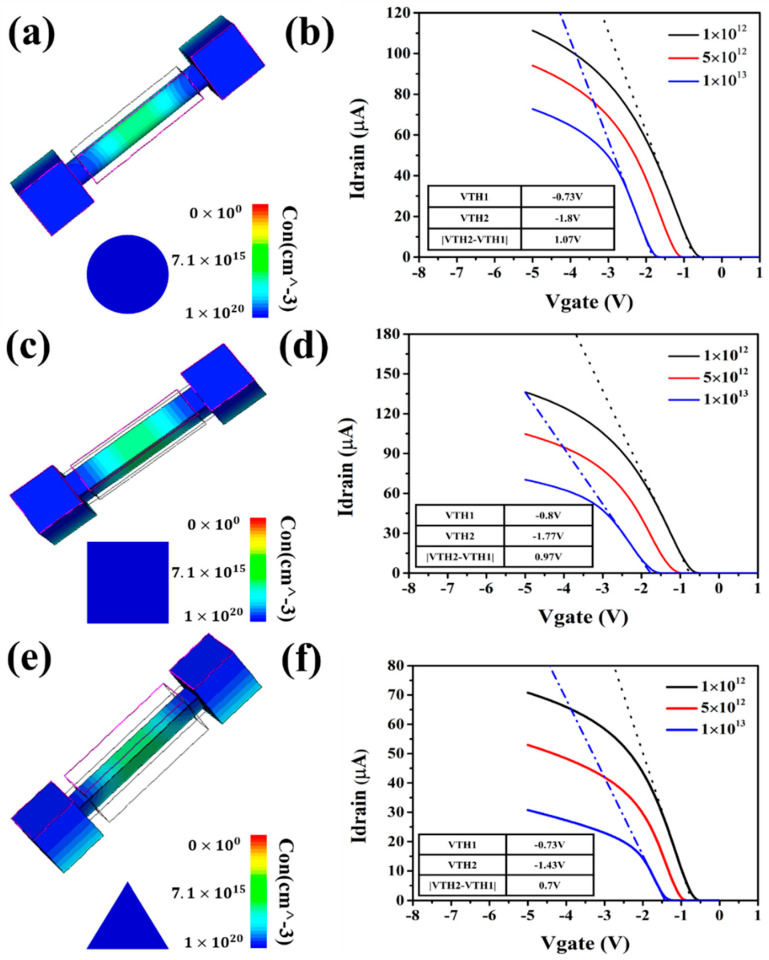
(**a**,**b**) Simulation results of SiNWs-FET with circle-cross-section; (**c**,**d**) Simulation results of SiNWs-FET with square cross-section; (**e**,**f**) Simulation results of SiNWs-FET with triangle cross-section.

Next, we used Sentaurus TCAD to simulate these three types cross-sectional SiNWs-FET sensors to verify our theoretical derivation. Charged target binding to a SiNWs-FET sensor will change its own threshold voltage. In Equation (2), the charge density *Qs* can be calculated by: $∆Q_{S}=C_{eq}\cdot ∆V_{TH}$. Therefore, the offset of the threshold voltage, obtained from the result of the simulation, can be used to support our theoretical analysis. We created three models of SiNWs-FET sensors with different cross-sections in Sentaurus TCAD, and set the lattice quality and the density of the interface state in turn. The value of the charge density was 1 × 10^12^, then 5 × 10^12^, and finally 1 × 10^13^, with the results of the simulation being recorded. The simulation results of SiNWs sensors with circular, square and triangular cross-sections are shown in Figure 4a,c,e, and the results of corresponding threshold voltage offset are shown in Figure 4b,d,f.

It is easy to obtain the equivalent transformation function of SiNWs-FET sensors’ sensitivity, which is given by

(8) $$sen\propto \frac{∆n}{n_{0}+n_{1}+n_{2}}=\frac{\frac{C_{eq}}{q}\cdot ∆V_{T}∆\frac{S_{surf}}{V}}{n_{0}+n_{1}+n_{2}}$$

The variation of carriers’ concentration is directly proportional to the drift of threshold voltage and surface-to-volume ratio. The surface-to-volume ratios of those three types of SiNWs-FET sensors are completely consistent with the results calculated from the geometric structure of SiNWs-FET sensor in Table 2. The offset of the threshold voltage is fully noted in Table 4.

Thus, we can obtain the following conclusion that $sen_{tri}>sen_{cir}>sen_{squ}$ using data in Table 2 and Table 4. To sum up, SiNWs-FET sensors with triangular cross-sections are more sensitive than the other two types of SiNWs-FET sensors when they have the same feature size. Moreover, the result calculated from Debye volume [38] is also in agreement with our analysis.

## 4. Conclusions

In this article, we proposed that the sensitivity of SiNWs-FET sensors is affected by several important factors, such as surface-to-volume ratio, depletion ratio, surface defects and quality of lattice. The sensitivity is given by:

(9) $$sen=\frac{∆I}{I_{0}}=\frac{\left(1+\frac{∆S}{S_{0}}\right)\cdot \left(Q_{S}\cdot \frac{S_{sur}}{V}\right)}{n_{0}+n_{1}+\int_{E_{SD}}^{E_{SD}^{\prime }}\frac{N_{SS}\left(E\right)dE}{1+\frac{1}{g}\cdot exp\left(\frac{E-E_{F}}{k_{0}T}\right)}}+\frac{∆S}{S_{0}}$$

Both the surface-to-volume ratio and the depletion ratio affect ∆*I*. The larger the surface-to-volume ratio and the larger the depletion ratio, the higher the sensitivity. Besides, both surface defects and lattice quality also have an impact on *I*_0_; the fewer the surface defects and the better quality of the lattice, the higher the sensitivity.

Our analysis indicates that many factors play important roles in the sensitivity of SiNWs-FET sensors. Under the same feature size, SiNWs-FET sensors fabricated by WETO with triangular cross-section are more sensitive than those sensors with circular or square cross-sections fabricated by other methods. Both the geometrical effect and the manufacturing process are extremely important factors for the performance of SiNWs-FET sensors.

## Figures and Tables

**Table 1:** The cross-sectional areas of three kinds of SiNWs-FET sensors.

Cross-Sectional Areas	Value
$S_{sec}^{tri}$	$\frac{\sqrt{3}}{4}W^{2}$
$S_{sec}^{squ}$	$W^{2}$
$S_{sec}^{cir}$	$\frac{\pi }{4}W^{2}$

**Table 2:** The surface-to-volume ratio of three types of SiNWs-FET sensors.

The Surface-to-Volume Ratio	Value
${\left(\frac{S_{sur}}{V}\right)}_{tri}$	$\frac{4\sqrt{3}}{w}$
${\left(\frac{S_{sur}}{V}\right)}_{squ}$	$\frac{4}{w}$
${\left(\frac{S_{sur}}{V}\right)}_{cir}$	$\frac{4}{w}$

**Table 3:** The depletion ratio of three kinds of SiNWs-FET sensors.

Depletion Ratio	Value
${\left(\frac{∆S_{sec}}{S_{sec}}\right)}_{cir}$	$1-\frac{{\left(W-2h\right)}^{2}}{W^{2}}$
${\left(\frac{∆S_{sec}}{S_{sec}}\right)}_{squ}$	$1-\frac{{\left(W-2h\right)}^{2}}{W^{2}}$
${\left(\frac{∆S_{sec}}{S_{sec}}\right)}_{tri}$	$1-\frac{{\left(W-2\sqrt{3}h\right)}^{2}}{W^{2}}$

**Table 4:** The offset of threshold voltage of three types of SiNWs-FET sensors.

The Offset of Threshold Voltage	Value
*∆V_TH-tri_*	0.7 V
*∆V_TH-squ_*	0.97 V
*∆V_TH-cir_*	1.07 V
